# Superconformal Electrodeposition of Cobalt into Micron-Scale Trench with Alkynol Derivatives

**DOI:** 10.3390/ma18081747

**Published:** 2025-04-10

**Authors:** Wei Xu, Yedi Li, Tingjun Wu, Yu Duan, Lei Zhu, Qiang Liu, Yiying Wang, Wenjie Yu

**Affiliations:** 1 School of Materials and Chemistry, University of Shanghai for Science and Technology, Shanghai 200093, China; 223353237@st.usst.edu.cn (W.X.); duanyu@mail.sim.ac.cn (Y.D.);; 2 National Key Laboratory of Materials for Integrated Circuits, Shanghai Institute of Microsystem and Information Technology, Chinese Academy of Sciences, Shanghai 200050, China; liyedi@mail.sim.ac.cn (Y.L.); leizhu@mail.sim.ac.cn (L.Z.);; 3 College of Materials Science and Opto-Electronic Technology, University of Chinese Academy of Sciences, Beijing 100049, China

**Keywords:** electrochemistry, cobalt interconnect, aldehyde-derived compounds, superfilling, adsorption energy, crystal orientation

## Abstract

Copper interconnect technology faces limitations due to the electron’s mean free path and electromigration, driving the adoption of cobalt alternatives. This study proposes a novel mechanism to achieve superfilling by tuning the adsorption energy of additive molecules on cobalt surfaces. The adsorption energies of additives are tailored by changing molecular structures with different functional groups. Computational results reveal that carbon–carbon triple bonds critically strengthen adsorption, while ether bonds further enhance binding on distinct cobalt crystallographic planes. Specifically, 1,4-bis(2-hydroxyethoxy)-2-butyne (BEO) containing both triple bonds and ether groups exhibits the highest adsorption energy (−22.62 eV). Replacing ether with hydroxyl groups in 2-butyne-1,4-diol (BOZ) reduces the adsorption energy to −10.39 eV, while eliminating triple bonds in 1,4-butanediol diglycidyl ether (BDE) further decreases it to −8.43 eV. Experimental studies demonstrate that BOZ and BEO preferentially adsorb on the (101) and (110) planes of hexagonal close-packed cobalt (HCP-Co) due to their differential adsorption energies. This selective suppression promotes preferential growth along the densely packed (002) orientation. This leads to a trench-filling process dominated by the most densely packed plane, resulting in better electrical performance. Superfilling is achieved when molecular adsorption energies are in the range of 5–8 eV. The work establishes a functional group design strategy to regulate additive adsorption, enabling crystallographic control for advanced cobalt electrodeposition processes.

## 1. Introduction

Copper is the conventional material for metal interconnect in very large scale integration (VLSI). However, as the process node develops from 180 nm to a remarkable 7 nm [1,2], interconnect sizes continue to shrink, resulting in high resistance and high resistance–capacitance (RC) delay [3,4,5]. The reason is that copper has a relatively large electron mean free path (39 nm) and needs a relatively thick barrier layer to inhibit electron migration [6,7]. Cobalt is a promising alternative material with an average electron free path of approximately 10 nm and low electron migration [8,9]. Therefore, at the same nanometer scale, cobalt has lower resistance and smaller RC delay [10,11]. The key to replacing copper with cobalt is to be able to produce high-quality cobalt interconnects.

Thin film deposition technology is critical to realize high-quality cobalt interconnects. Physical vapor deposition [12,13] (PVD), chemical vapor deposition [14,15] (CVD), atomic layer deposition [16,17,18] (ALD), and electrodeposition [19,20,21] are the primary synthesis methods reported to date. PVD is a widely used film deposition technique, yet its filling efficiency and step coverage remain inadequate, primarily due to one-direction linear deposition characteristics. This results in particles with large incidence angles failing to enter the trenches and instead depositing at the trench openings [22]. Consequently, the opening closes before the trench is fully filled [23]. To solve this problem, the industrial sector has developed specific PVD technologies, such as ionized PVD and collimated sputtering [24,25]. Nevertheless, the hole problem in small-sized channels still exists. In addition, CVD technologies have better filling capabilities, especially when trench widths are around several hundred nanometers, such as plasma-enhanced chemical vapor deposition [26]. ALD possesses the unique characteristic of layer-by-layer self-limiting growth, providing exceptional step coverage and film uniformity. However, its deposition rate is exceedingly low, making it particularly suitable for applications such as barrier layers and seed layers [27]. In contrast, electroplating technology is extensively utilized in damascene copper interconnect processes, where it enables bottom-up film growth and demonstrates remarkable filling capabilities [28,29]. During the electroplating process, precise control over parameters such as applied current, anode material, accelerants, inhibitors, planarization agents, and mass transport allows for the fine-tuning of deposition rate, grain size, morphology, impurity content, and stress [30]. This level of control highlights the considerable potential for further advancement of metal-interconnect trench-filling techniques.

Cobalt electroplating is an efficient and cost-effective method for achieving high-quality cobalt interconnects [31,32]. By introducing certain organic additives into the plating solution, bottom-up cobalt superfilling can be realized. Superfilling refers to the phenomenon where the metal deposition rate at the bottom of a trench exceeds that in the side wall. This technique is particularly crucial for the fabrication of interconnects with high aspect ratios, as it effectively prevents the formation of voids, ensuring the integrity and reliability of the interconnects. To date, multiple super-conformal filling mechanisms have been proposed by different research groups. The S-shaped negative differential resistance [33,34] has been extensively applied to the overfilling processes of various metals, including copper [35], gold [36], nickel [37], cobalt [38], and zinc [39]. This model suggests that the adsorption and desorption behavior of inhibitors on the electrode surface leads to a nonlinear variation in current density within a specific potential range, resulting in an “S”-shaped curve. By optimizing inhibitor concentration and potential control, this model enables the realization of the bottom-up overfilling of metals in high-aspect-ratio structures. Rigsby et al. [40,41] introduced the differential current efficiency fill mechanism, emphasizing the critical role of the H^+^ concentration gradient in overfilling processes. They observed that during the electrodeposition of cobalt, the pH gradient rapidly formed within the trenches due to proton transfer, resulting in a disparity between the current efficiency and deposition rate at the trench interior and the opening. This differential effect facilitated the bottom-up filling of cobalt. In parallel, Wu et al. [42] introduced the hydrogen reduction-induced deactivation mechanism, which elaborates on how changes in pH and H^+^ concentration affect the inhibitory efficacy of additives, thereby triggering a bottom-up filling mechanism. That model showed that the self-initiated bottom-up filling of cobalt was driven by the reduction in and deactivation of inhibitors at the bottom of the trench, caused by hydrogen gas evolution, leading to a diminished inhibitory effect and subsequently promoting the deposition rate at the bottom. The adsorption energy determines the conformality of deposited cobalt layers, especially in complex geometries’ structures, but its role in achieving superconformal filling has been largely overlooked. Addressing this gap is essential for improving the precision and reliability of coating processes in advanced applications.

In this paper, we propose a mechanism to achieve superfilling by regulating the adsorption energy of molecules on the cobalt surface. By rationally designing the chemical structure of the additive molecules (such as introducing specific functional groups or adjusting the molecular size), the adsorption energy of molecules on the cobalt surface can be regulated. It is worth noting that alkynol additives [43,44,45,46] (such as 2-butyne-1,4-diol) exhibit a strong adsorption effect on the (100) and (101) crystal planes of hexagonal close-packed (HCP) cobalt, which provides a potential way to regulate the adsorption energy of molecules on the cobalt surface. However, at present, there is still a lack of in-depth research on the filling effect of alkynols in cobalt interconnect trenches and the regulation mechanism of specific groups on the adsorption energy of molecules. We investigate the effects of three organic additives—2-butyne-1,4-diol (BOZ), 1,4-bis(2-hydroxyethoxy)-2-butyne (BEO), and 1,4-butanediol diglycidyl ether (BDE)—on the morphology, structure, and electrical properties of cobalt electroplating. BEO, which adds two ether groups compared to BOZ, and BDE, which lacks the triple bond and hydroxyl group found in BOZ and BEO, to highlight the role of the alkyne–ether functionality and clarify the influence of the unsaturated triple bond. Through electrochemical experiments, we compare the polarization capabilities of these additives and analyze their behavior during the electroplating process. By calculating the surface energies of different crystal planes, we uncover the mechanism behind selective crystal facet adsorption. Using quantum chemical methods, we further examine the adsorption and desorption behaviors of organic molecules during the electroplating process.

## 2. Materials and Methods

### 2.1. Materials

The basic plating solution is a cobalt sulfate–boric acid system consisting of 0.05 mol/L heptahydrate cobalt sulfate (CoSO_4_·7H_2_O, 99.99%, Shanghai Aladdin Biochemical Technology Co., Ltd., Shanghai, China) and 0.485 mol/L boric acid (H_3_BO_3_, 99.99%, Shanghai Aladdin Biochemical Technology Co., Ltd., Shanghai, China). The additives include BOZ (C_4_H_6_O_2_, 99%, Shanghai Aladdin Biochemical Technology Co., Ltd., Shanghai, China), BEO (C_8_H_14_O_4_, 95%, Shanghai Macklin Biochemical Technology Co., Ltd., Shanghai, China), and BDE (C_10_H_18_O_4_, 95%, Shanghai Aladdin Biochemical Technology Co., Ltd., Shanghai, China). Unless otherwise specified, the concentration of each additive mentioned in the text is 50 mg/kg.

The electrochemical experimental setup used was a multi-channel electrochemical workstation (Model M204) manufactured by Metrohm (Herisau, Switzerland). A standard three-electrode system was employed, with a Pt electrode (Tianjin Aida Hengsheng Technology Development Co., Ltd., Tianjin, China) as the counter electrode, a saturated Ag/AgCl electrode (Tianjin Aida Hengsheng Technology Development Co., Ltd., Tianjin, China) as the reference electrode, and a Co/TiN/Si patterned wafer (Prepared by magnetron sputtering) as the working electrode. The patterned wafer had a line width of 3 μm and a trench depth of 1 μm. Cobalt electrodeposition was performed using the constant current chronopotentiometry method. During deposition, only one square surface was exposed, while the remaining area was tightly clamped with the Pt electrode, deposited at a current density of 3 mA/cm^2^ for 56.5 min. Linear sweep voltammetry (LSV) was performed from 0 to −1.0 V vs. Ag/AgCl using a negative potential scan at a rate of 2 mV·s^−1^. The stability of the additives during electrodeposition was evaluated in an acidic solution (pH 4.00) of 30 g/L H_3_BO_3_ (See Figure S1 for details).

### 2.2. Methods

#### 2.2.1. Calculation Details

The quantum chemical calculations were performed using Gaussian 16 W (Version 1.1) with the B3LYP [31] method in density functional theory (DFT). Geometry optimizations were carried out at the 6–311 G basis set level. The energies of HOMO, LUMO, and their energy gap were investigated. Additionally, Multiwfn (Version 3.8) was utilized to [47,48] analyze the electrostatic potential (ESP) distribution map and the average local ionization energy, which were used to evaluate the adsorption capability of the additives on the metal surface and to identify reactive sites.

To investigate the adsorption interactions between additives and the Co surface, all density functional theory (DFT) calculations, including total energy computations and geometry optimizations, were conducted using the projector augmented-wave method and generalized gradient approximation potentials with the Perdew–Burke–Ernzerhof exchange-correlation functional, as implemented in the Vienna Ab initio Simulation Package. To account for long-range interactions, the DFT-D3 dispersion correction method of Grimme was employed. The plane-wave energy cut-off was set to 450 eV, ensuring convergence of bulk energies within 0.001 eV/atom. Cobalt is known to exist in two crystalline phases: the face-centered cubic (FCC) phase and the hexagonal close-packed (HCP) phase. In our experiment, cobalt predominantly adopts the HCP phase. Accordingly, the slab models were based on the most stable HCP (002) surfaces, represented by periodic slabs comprising four-layer 6 × 6 supercells with a vacuum layer of 20 Å to ensure accurate surface energy calculations. Monkhorst–Pack k-point sampling of 2 × 2 × 1 was applied. During structural optimization, the adsorbate molecule and the two topmost surface layers were allowed to relax, while the remaining slab atoms were kept fixed. The interaction energy ($∆E_{ad}$) was calculated as:

(1) $$∆E_{ad}=E_{complex}-(E_{surface}+E_{additive})$$

where $E_{complex}$, $E_{surface}$, and $E_{additive}$ are the total energy of the optimized co-additive complex, cobalt surface, and the isolated additive molecule, respectively. Ovito version 3.10.6 was used to produce the snapshot pictures of all simulations.

#### 2.2.2. Material Characterization

The crystalline structure of the cobalt thin film after deposition was characterized using X-ray diffraction (XRD, Bruker D8 Discover, Bruker, Berlin, Germany). The X-ray source was a Cu rotating anode target, with a tube voltage and current of 40 kV and 450 mA, respectively. The scanning range was 30° to 90° with a step size of 0.02°. The bulk resistivity of the film was measured using a four-point probe system (KEITHLEY 4200A-SCS, Keithley Instruments, Cleveland, OH, USA), with an average value obtained from nine measurement points. The thickness and cross-sectional morphology of the film were observed using a field-emission scanning electron microscope (FESEM, JSM-7800F, JEOL, Tokyo, Japan). The resistivity of the film was determined by combining the film thickness and sheet resistance. The details of cobalt filling in the grooves were observed using a focused-ion-beam scanning electron microscope (FIB-SEM, Zeiss crossbeam 350, Carl Zeiss AG, Oberkochen, Germany), and the arrangement of cobalt atoms in the grooves was analyzed by a field-emission transmission electron microscope (TEM, JEM-F200, JEOL, Tokyo, Japan). The cobalt film outside the trenches was removed through mechanical polishing or chemical mechanical polishing (CMP), retaining only the cobalt interconnects within the trenches. Given the small dimensions of the test structure (typically 1000 × 1000 μm^2^), a microscope-integrated manual probe station was employed to precisely position probes onto the predefined top electrodes. Resistance measurements were then performed using a Keithley 2400 SourceMeter (Keithley Instruments, Cleveland, OH, USA) to acquire accurate resistance values. The line resistivity of the interconnects was then calculated based on the cross-sectional area and length of the interconnects.

## 3. Results and Discussion

### 3.1. Molecular Activity Analysis

**Figure 1:**
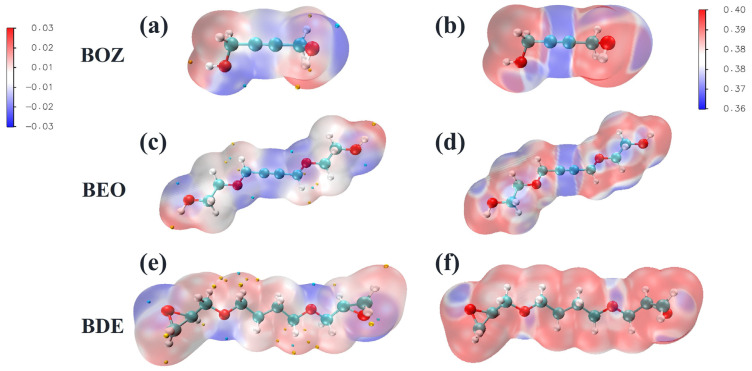
Electrostatic potential and average local ionization energy (ALIE): (**a**,**b**) BOZ, (**c**,**d**) BEO, (**e**,**f**) BDE.

To clarify the molecular activity and reaction sites of the three additives, BOZ, BEO, and BDE, the DFT was utilized to investigate the molecular electronic structures and orbital energies. Table 1 presents the *E_HOMO_*, *E_LUMO_*, and the energy gap (Δ*E* = *E_LUMO_* − *E_HOMO_*) of BOZ, BEO, and BDE molecules. Generally, for molecules with similar structures, a higher *E_HOMO_* and lower *E_LUMO_* indicate a greater ability to donate electrons to the unoccupied d-orbitals of the transition cobalt metal and a greater ability to accept electrons, respectively. A smaller ΔE suggests a more stable adsorption of the molecule on the cobalt surface [49]. Comparing the *E_HOMO_* values of the studied molecules, it was evident that BEO had a significantly higher *E_HOMO_*, indicating a stronger electron-donating ability. Furthermore, the ΔE values of BOZ and BEO were significantly lower than that of BDE, suggesting that BOZ and BEO, with carbon–carbon triple bonds, had a greater potential for adsorption on the Co surface. Figure 1 shows the ESP maps and the average local ionization energy (ALIE) surface distributions of the BOZ, BEO, and BDE molecules, aiming to identify their preferred adsorption sites. The ESP reflects the ability of molecules to approach each other through electrostatic attraction in the early stages of a chemical reaction. The ALIE distribution illustrates the electronic activity of two molecules as their van der Waals surfaces come into close proximity. Figure 1a,c,e show the ESP maps of the studied molecules. The blue regions represent electron-rich areas, while the red regions indicate electron-deficient areas. Clearly, for the BOZ and BEO molecules, the space around the triple-bonded carbon and oxygen atoms exhibited higher electron density. In the BDE molecule, the four oxygen atoms displayed higher electron density. Figure 1b,d,f show the surface distribution of the average local ionization energy for the studied molecules. The blue regions indicate areas with lower ALIE values, signifying stronger electronic activity. For the BOZ and BEO molecules, the regions around the triple-bonded carbon and oxygen atoms, and for the BDE molecule, the regions surrounding the four oxygen atoms corresponded to lower ALIE values. Therefore, the triple-bonded carbon and oxygen in BOZ and BEO, as well as the oxygen atoms in BDE, were identified as active sites, making them prone to interacting with the Co surface.

### 3.2. Electrochemical Analysis

**Figure 2:**
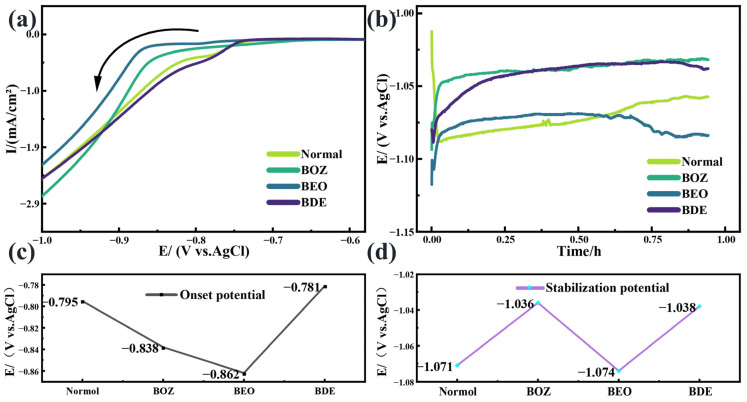
(**a**) Linear sweep voltammetry (LSV) curves, arrows are scanning directions, (**b**) chronoamperometric deposition potential curves for cobalt filling, (**c**) initial deposition potentials for cobalt filling, and (**d**) stabilization deposition potentials for cobalt filling with different additive.

The electrochemical behavior during cobalt electrodeposition was analyzed using linear sweep voltammetry (LSV) and the electrochemical deposition method with constant current. The LSV test involved a negative sweep starting from 0 V to −1 V vs. Ag/AgCl with a scan rate of 2 mV·s^−1^, as shown in Figure 2a. The results indicated that after adding BOZ and BEO, the onset potential for the increase in current density shifted negatively (the potentials of the control group at −0.795 V shifted negatively to −0.838 V and −0.862 V, as shown in Figure 2c). This implies that cobalt deposition was suppressed at the same applied potential, with BEO exhibiting a stronger inhibitory effect than BOZ. The inhibition of cobalt deposition is attributed to the formation of an adsorption layer on the electrode surface by the additives, which alters the electrode properties and increases the energy barrier for cobalt deposition on the substrate [50]. In contrast, no negative shift was observed in the presence of BDE, indicating that the adsorption capability of BDE on the substrate was weaker than that of BOZ and BEO. This highlighted the significant contribution of the carbon–carbon triple bond to enhance the adsorption energy of the additives, which was consistent with the DFT analysis results discussed in Section 3.1.

Figure 2b shows the galvanostatic chronopotentiometric cobalt deposition curve on a Co/TiN/Si substrate with different additives. The reaction was conducted at a current density of 3 mA/cm^2^. The major side-reaction is the hydrogen evolution reaction (HER), as the standard electrode potential of H^+^ is 0 V, while that of Co^2+^ is −0.277 V. With a more positive potential, H^+^ is more likely to be reduced than Co^2+^, as shown in reactions (2) and (3). Consequently, H_2_ generation is inevitable during cobalt deposition, which often increases the local OH^−^ concentration on the cobalt surface [51], leading to the formation of by-products [52,53]. The side reactions are represented by reactions (4) and (5).

(2) $${2H}^{+}+2e^{-}\to H_{2}|E_{0}=0\;V$$

(3) $${Co}^{2+}+2e^{-}\to Co|E_{0}=-0.277\;V$$

(4) $${Co_{\left(aq\right)}}^{2+}+{2{OH}_{\left(aq\right)}}^{-}\to Co{\left(OH\right)}_{2\left(s\right)}$$

(5) $$Co{\left(OH\right)}_{2\left(s\right)}+2e^{-}\to Co_{\left(s\right)}+2O{H_{\left(aq\right)}}^{-}$$

It is well known that during the early stages of electrochemical deposition, especially on the cathode surface, a high cathodic overpotential, a large total number of adsorbed atoms, and the low surface mobility of adsorbed atoms are essential conditions for nucleation. The presence of a cobalt seed layer significantly reduces the nucleation requirements, initiating cobalt deposition almost immediately upon applying the current in the absence of additives. However, the adsorption of additives necessitates a certain overpotential to break through the adsorbed layer [54], where the potential gradually decreases and stabilizes. The stabilization deposition potential generally reflects the cathodic polarization ability of the additives. The polarization induced by BOZ and BDE shifted the potential positively (the potentials of the control group at −1.071 V shifted negatively to −1.036 V and −1.038 V, respectively, as shown in Figure 2d). Furthermore, a positive potential shift implies reduced energy consumption in the system. According to reactions (2) and (3), the energy required to reduce Co^2+^ is greater than that for H^+^. Under constant current density, the amount of charge exchanged within a certain period of time is fixed. Thus, when cobalt deposition is suppressed, a positive potential shift indicates the promotion of the hydrogen evolution reaction. In the case of BEO, this positive potential shift was limited, suggesting that the hydrogen evolution reaction was less intense than with BOZ, which may be attributed to the introduction of ether groups.

### 3.3. Morphology and Crystalline Structure Analysis

**Figure 3:**
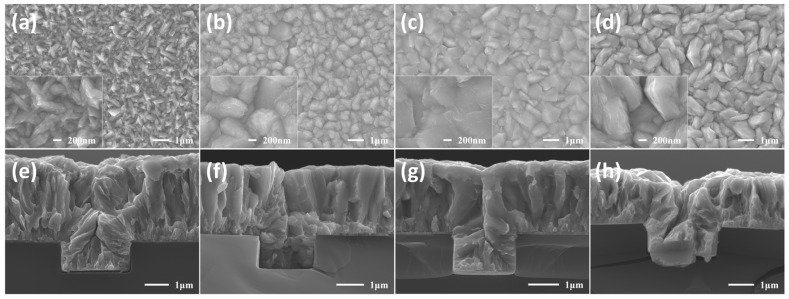
Surface SEM images of the electrodeposited cobalt in solution (**a**) without additives, with (**b**) BOZ, (**c**) BEO, (**d**) BDE. Cross-sectional SEM images of 1 μm trenches deposited in an electrolyte (**e**) without additives and (**f**) BOZ, (**g**) BEO, (**h**) BDE.

To investigate the effect of additives on crystal morphology, SEM characterization was performed on the surface and cross-sectional morphology of cobalt films after electrodeposition. As shown in Figure 3, the surface and cross-sectional morphologies of the cobalt films are presented. The super-conformal filling was achieved in the electrolyte with BDE in micro-scale trend. The cross-sectional of Co deposited with the addition of BOZ and BEO showed a gap, although the gap already shrunk compared to the one without additives. It is evident that prolonged electrodeposition allowed sufficient time for the cobalt grains to grow, and the addition of additives significantly altered the morphology of the deposited Co. When Co was deposited in the solution without additives (as shown in Figure 3a), the presence of cobalt dendrites [55] was observed. Under the influence of the tip effect, these cobalt dendrites may lead to the premature narrowing of openings. The addition of organic additives effectively suppressed the formation of dendrites. The cobalt grains became larger and more uniform with the presence of BOZ, BEO, and BDE. Specifically, BEO also made the surface of the cobalt film more compact, but the grain contours became more blurred. This might be owing to the effect of the ether bonds. In the cross sections shown in Figure 3e–h, no significant filling defects were observed. However, the cleavage planes of the BOZ-treated sample (Figure 3f) and the BEO-treated sample (Figure 3g) appeared much smoother compared to the control group (Figure 3e) and the BDE-treated sample (Figure 3h).

**Figure 4:**
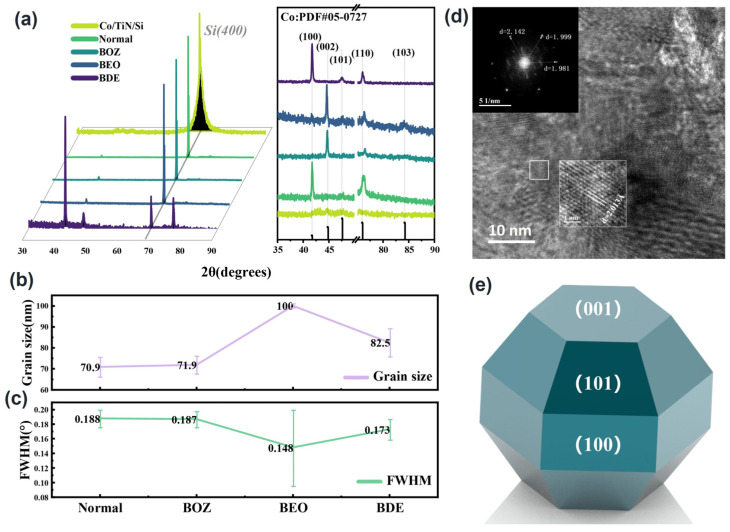
XRD patterns of channel-deposited cobalt obtained from electroplating solutions with different additives: (**a**) XRD spectra of channel cobalt (left: full spectrum, right: spectrum with substrate peaks removed), (**b**) grain size of the main cobalt peak, (**c**) FWHM of the main cobalt peak, (**d**) TEM image of the BEO-filled cross section, and (**e**) schematic diagram of the crystal cell.

The effect of organic additives on the crystal structure was further investigated by XRD. Figure 4a reveals that cobalt growth in the trench covered with PVD Co as a seed layer was well crystallized. Due to the intense peak of the silicon substrate (100) plane at 69.1°, the right panel of Figure 4a only shows the peak profiles in the ranges of 35–50° and 75–90°. During cobalt electrodeposition, cobalt preferentially grew along the (100) direction with strong crystallinity in the electrolyte without additives and with BDE. However, in the electrolyte with BOZ or BEO, the preferred orientation shifted to the (002) direction. Additionally, the (002) crystal orientation of Co deposited with BEO was also observed in the TEM image (Figure 4d). The change in the Co preferential orientation may be related to the specific adsorption of additives on certain crystal planes. This adsorption inhibits the growth of the adsorbed planes, causing the crystal to preferentially grow along the non-adsorbed surfaces.

The grain size of cobalt was estimated using the Scherrer formula based on the Full Width at Half Maximum (FWHM) of cobalt, as shown in Figure 4b,c. The grain size of the cobalt film with BOZ was slightly larger than that of the control group without additives. In contrast, the addition of BDE caused a significant increase in grain size. The average grain size of the cobalt film with BEO was larger than 100 nm, at which point the Scherrer formula was no longer accurate for calculating grain size. However, it demonstrated that BEO could enlarge the grain size significantly. The FWHM value reflects the internal micro-strain and crystal quality of the material. As shown in Figure 4c, the cobalt films under the influence of BEO and BDE exhibited better quality than those in the control group and BOZ, with an overall superior crystallinity which aligned with the SEM results.

Table 2 lists the relative texture coefficients (RTC) of the (100), (002), and (101) crystallographic planes in cobalt thin-film samples. The relative texture coefficient (RTC) is an important parameter used to quantify the preferred orientation of grains in materials. It is defined as the ratio of the diffraction intensity of a specific crystallographic plane (hkl) to the average diffraction intensity of all measured planes. The RTC value can be used to evaluate the relative orientation degree of a specific plane in the material and its influence on crystal growth behavior. The calculation formula for RTC is shown in Formula (6):

(6) $${RTC}_{\left(hkl\right)}=\frac{I_{\left(hkl\right)}/I_{\left(hkl\right)}^{0}}{\sum_{1}^{n}I_{\left(hkl\right)}/I_{\left(hkl\right)}^{0}}\times 100\%$$

Here, $I_{\left(hkl\right)}$ represents the experimentally measured diffraction intensity of the specific crystallographic plane (hkl), and $I_{\left(hkl\right)}^{0}$ denotes the expected diffraction intensity of this plane in a completely randomly oriented material, typically obtained from standard samples.

Experimental data indicated that in the additive-free control group, the crystal orientation of the cobalt thin film was (100), with an RTC_(100)_ value as high as 95%. However, upon the addition of BOZ and BEO additives, the preferred growth orientation of the cobalt thin film shifted from the (100) to the (002) orientation. For the samples with BOZ and BEO additives, the RTC_(002)_ values reached 79% and 59%, respectively, representing increases of 30.5 times and 22.8 times compared to the RTC_(002)_ value of the control group (2.6%). This notable orientation transition is likely closely related to the presence of carbon–carbon triple bonds (C≡C) in the BOZ and BEO molecules. A theoretical analysis suggests that the carbon–carbon triple bonds may strongly adsorb onto the (001) and (101) planes of the cobalt thin film, thereby inhibiting the growth of these planes and promoting the (002) plane as the dominant growth orientation.

To further validate this hypothesis, the effect of the BDE molecule, which lacks a carbon–carbon triple bond, on the crystal orientation of the cobalt thin film was also investigated. Experimental results showed that under the influence of BDE, the RTC_(002)_ value of the cobalt thin film was only 0.34%, significantly lower than that observed with BOZ and BEO additives. Although the BDE molecule retains an ether group (C-O-C), its lack of a carbon–carbon triple bond structure did not significantly promote the preferred growth of the (002) plane. This phenomenon indicated the critical role of the carbon–carbon triple bond in inducing the transition in crystal orientation. In summary, the carbon–carbon triple bond altered the crystal growth direction of the cobalt thin film by adsorption onto specific crystallographic planes, leading to the preferred orientation of (002).

### 3.4. Electrical Performance Analysis

**Figure 5:**
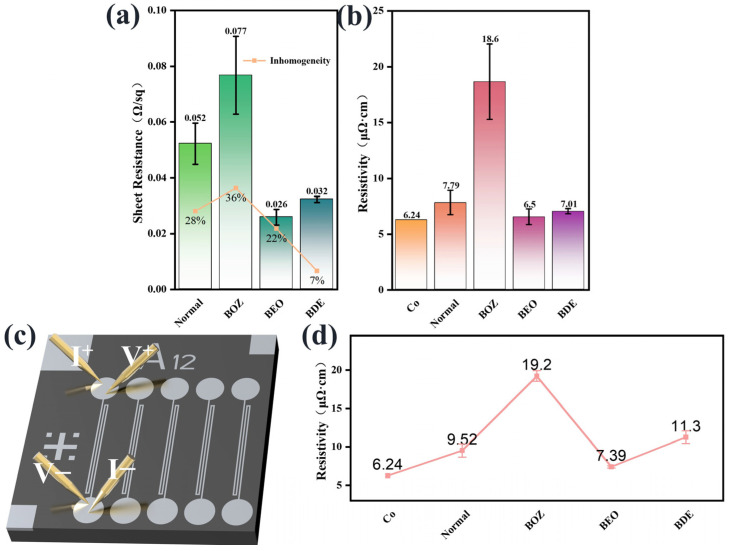
The electrical properties of electroplated cobalt under different additive conditions. (**a**) Sheet resistance of cobalt films. (**b**) Bulk resistivity of cobalt films. (**c**) Schematic diagram of the four-wire resistance method. (**d**) Line resistivity of filled cobalt.

The sheet resistance of the cobalt films was measured using the four-point probe method to investigate the effect of organic additives on the electrical performance of deposited cobalt. Typically, the sheet resistance of a thin film is influenced by its thickness and resistivity, and the resistivity is affected by factors such as crystal structure, dislocations, defects, and impurities. Since the sheet resistance was measured at nine different locations on the cobalt film, the fluctuations in the measured values reflected the uniformity of the film properties. The non-uniformity of the film properties was quantified by the ratio of the difference between the maximum and minimum sheet resistance values to the average value ((Max − Min)/Avg). Figure 5a shows that compared to cobalt films deposited without additives, the addition of BOZ increased the non-uniformity of the cobalt film, while the addition of BEO and BDE effectively controlled the non-uniformity.

Using Equation (7), the resistivity of the cobalt film can be calculated, where *ρ* is the resistivity, *d* is the film thickness, and *R_s_* is the sheet resistance. The film thickness was determined by the SEM cross-sectional image, as shown in Figure 5b. The resistivity of bulk cobalt is 6.24 μΩ·cm. The higher resistivity of electrodeposited Co films is primarily attributed to lattice distortion and electron scattering. Notably, cobalt films deposited using BOZ-containing solutions showed higher resistivity values compared to other samples. According to SEM images, this may be caused by the non-uniformity and loose grain structure of the cobalt films. In contrast, cobalt films deposited with BEO demonstrated superior electrical performance compared to those deposited with BOZ or BDE and exhibited a resistivity value comparable to that of the annealed cobalt films reported by Natalia et al. [56], benefiting from their compact grain structure.

(7) $$\rho =d\cdot R_{s}$$

In the interconnection technology of microchips, the line resistivity of the interconnection material increases rapidly as the interconnection size decreases, and this phenomenon has a significant impact on the performance of the chip. Therefore, it was necessary to further study the electrical properties of cobalt filled in silicon trenches. We used advanced CMP technology to remove the excess cobalt film on the surface of the patterned silicon wafer to ensure more accurate resistivity measurement. The line resistivity of cobalt line (Figure 5d) was measured by the four-probe method (Figure 5c). The line resistivity of the BEO sample increased by 13.7% compared with the film resistivity. Additionally, the resistivity of the BOZ sample also revealed a similar phenomenon, and its line resistivity increased relative to the bulk resistivity.

### 3.5. Selective Growth of Cobalt

**Figure 6:**
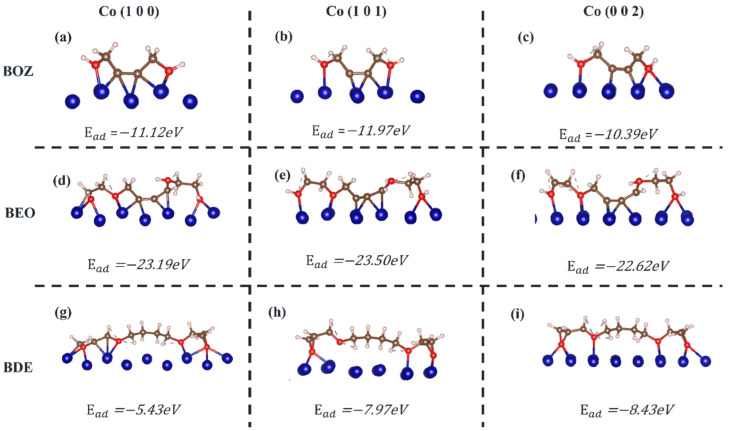
The adsorption capacity of additives on different surfaces. (**a**) BOZ adsorbed on cobalt (100) crystal surface. (**b**) BOZ adsorbed on cobalt (101) crystal surface. (**c**) BOZ adsorbed on cobalt (002) crystal surface. (**d**) BEO adsorbed on cobalt (100) crystal surface. (**e**) BEO adsorbed on cobalt (101) crystal surface. (**f**) BEO adsorbed on cobalt (002) crystal surface. (**g**) BDE adsorbed on cobalt (100) crystal surface. (**h**) BDE adsorbed on cobalt (101) crystal surface. (**i**) BDE adsorbed on cobalt (002) crystal surface.

To further elucidate the selective adsorption capabilities of the BOZ, BEO, and BDE molecules on different crystal planes of the Co surface in the pattern, we conducted DFT computational simulations of the surface energies of the (101), (100), and (002) Co surfaces, as well as the adsorption behaviors of the three molecules on these Co surfaces. The adsorption conformations and adsorption energies are shown in Figure 6. All molecules tended to adsorb on the Co surface in a parallel manner through their reactive atoms. Consequently, the BEO molecule, which had the most reactive atoms, exhibited a lower adsorption energy on the Co surface compared to BOZ and BDE, indicating the strongest adsorption capability. The adsorption conformations of BOZ and BEO on the Co surface exhibited a zigzag pattern. In contrast, BDE formed bonds with Co only at its two terminal oxygen atoms, leaving the four central carbon atoms unbound to Co. As a result, the inhibition film formed by BDE on the Co surface was looser compared to those formed by BOZ and BEO. To analyze the selective adsorption of molecules on different crystal planes, we first examined the BOZ and BEO molecules. As shown in Figure 6a–f, the BOZ and BEO molecules exhibited stronger adsorption on the (100) and (101) planes, as indicated by the lower adsorption energy. This suggests that the growth of the (100) and (101) crystal planes was suppressed, while the (002) crystal plane began to grow. Furthermore, we observed that the C≡C bond length stretched from 0.12 nm before adsorption to 0.137 nm, aligning with the C=C bond length in ethylene molecules. This indicated that the triple-bonded carbon atoms in BOZ and BEO had transitioned from sp hybridization to sp² hybridization after adsorption [57], forming strong chemisorption interactions with the Co surface. In cobalt deposition without additives, the (101) crystal plane preferentially grew with the highest growth rate. When the BDE additive was introduced, due to the strongest adsorption inhibition effect of BDE on the (002) crystal plane (E_ad_ = −8.43 eV), Co still preferentially grew on the (101) and (100) crystal planes.

## 4. Conclusions

In this study, a superfilling mechanism was proposed, in which superfilling was achieved by regulating the adsorption energy of molecules on the cobalt surface. Organic additives containing carbon–carbon triple bonds and oxygen-containing functional groups (BOZ, BEO, and BDE) were examined. The molecular activity and electrochemical behavior analysis revealed that BOZ and BEO exhibited stronger adsorption capabilities on the cobalt surface compared to BDE, owing to their carbon–carbon triple bonds. XRD results indicated that after the addition of BOZ, BEO, and BDE, the cobalt grain size significantly increased. XRD results and adsorption energy calculations demonstrated that the strong adsorption of BOZ and BEO on the (100) and (101) planes of hexagonal close-packed cobalt (HCP_Co) promoted a preferred orientation along the (002) plane of HCP_Co. The adsorption energy of BOZ, BEO, and BDE were −10.39 eV, −22.62 eV, and −8.43 eV, respectively. Cobalt superfilling into micro-size trench was achieved by using BDE molecules as additives with an absorption energy of 8 eV. Additives with an extrahigh adsorption energy may inhibit the deposition rate of Co equally both on top and at the bottom of the trench, which cannot benefit from superfilling. However, additive molecules with a suitable adsorption energy may inhibit the Co reduction reaction more strongly on top of the trench than at the bottom, because of the additive molecule concentration gradient caused by mass transport and additive consumption during electrodeposition.

## Figures and Tables

**Table 1:** Electronic parameters of the three additives, including *E_HOMO_*, *E_LUMO_*, and the energy gap (Δ*E* = *E_LUMO_* − *E_HOMO_*).

Additive	HOMO (eV)	LUMO (eV)	ΔE (eV)
BOZ	−7.341	0.066	7.407
BEO	−6.874	0.825	7.698
BDE	−7.319	0.872	8.192

**Table 2:** Relative texture coefficients of each crystal surface of cobalt thin films under the action of different additives.

	I	I/I_0_	RTC_(100)_	I	I/I_0_	RTC_(002)_	I	I/I_0_	RTC_(101)_
PDF 05-0727	20	1	33%	60	1	33%	100	1	33%
Normal	843	42.1	95%	69	1.2	3%	118	1.2	3%
BOZ	61	3.1	17%	867	14.5	79%	80	0.8	4%
BEO	141	7.1	35%	719	12.0	59%	129	1.3	6%
BDE	2211	110.6	97%	23	0.4	0%	274	2.7	3%

## Data Availability

The original contributions presented in this study are included in the article. Further inquiries can be directed to the corresponding author.

## Supplementary Materials

The following supporting information can be downloaded at: https://www.mdpi.com/article/10.3390/ma18081747/s1, Figure S1: CVs of the additive in different solutions.

## References

[1] Simon A., van der Straten O., Lanzillo N.A., Yang C.-C., Nogami T., Edelstein D.C. (2020). Role of High Aspect-Ratio Thin-Film Metal Deposition in Cu Back-End-of-Line Technology. J. Vac. Sci. Technol. A.

[2] Edelstein D., Uzoh C., Cabral C., DeHaven P., Buchwalter P., Simon A., Cooney E., Malhotra S., Klaus D., Rathore H. A High Performance Liner for Copper Damascene Interconnects. Proceedings of the IEEE 2001 International Interconnect Technology Conference (Cat. No.01EX461).

[3] Kuo C.-Y., Zhu J.-H., Chiu Y.-P., Ni I.-C., Chen M.-H., Wu Y.-R., Wu C.-I. (2024). Graphene-All-Around Cobalt Interconnect with a Back-End-of-Line Compatible Process. Nano Lett..

[4] Zhang L., Wang T., Xie S., Lu X. (2023). The Role of Diethanolamine on Chemical Mechanical Polishing in Alkaline Glycine-Based Slurries for Cobalt Interconnects. ECS J. Solid State Sci. Technol..

[5] Lanzillo N.A., Chu A., Bhosale P., Dechene D. (2022). Power Delivery Design, Signal Routing, and Performance of On-Chip Cobalt Interconnects in Advanced Technology Nodes. IEEE Trans. Very Large Scale Integr. VLSI Syst..

[6] Wang J., Ke D., Tian F., Xiong L., Chen H., Li M. (2024). Co Contamination of Si: Plating & Aging. Heliyon.

[7] Mont F.W., Zhang X., Wang W., Kelly J.J., Standaert T.E., Quon R., Ryan E.T. (2017). Cobalt Interconnect on Same Copper Barrier Process Integration at the 7nm Node. Proceedings of the 2017 IEEE International Interconnect Technology Conference (IITC).

[8] Fang J.-S., Su T.-H., Cheng Y.-L., Huang C.-W., Chen G.-S. (2025). Influence of Trace Tungsten Contents on Thin-Film Properties and Electromigration Behaviors of Electroless-Deposited Cobalt Interconnect Lines. Mater. Res. Bull..

[9] Fang J.-S., Lee W., Cheng Y.-L., Lin C.-I., Chen G.-S. (2025). The Encaging of Cobalt Interconnect Lines with an Ordered Amino-Based Self-Assembled Monolayer for Electromigration Mitigation Using an All-Wet Electroless Process. Thin Solid Films.

[10] Kim C., Kang G., Jung Y., Kim J.-Y., Lee G.-B., Hong D., Lee Y., Hwang S.-G., Jung I.-H., Joo Y.-C. (2022). Robust Co Alloy Design for Co Interconnects Using a Self-Forming Barrier Layer. Sci. Rep..

[11] Zhou G., Sun T., Younas R., Hinkle C.L. Materials and Device Strategies for Nanoelectronic 3D Heterogeneous Integration. Proceedings of the 2021 International Conference on Simulation of Semiconductor Processes and Devices (SISPAD).

[12] Ichou H., Arrousse N., Berdimurodov E., Aliev N. (2023). Exploring the Advancements in Physical Vapor Deposition Coating: A Review. J. Bio Tribo Corros..

[13] He Y., Mao S., Xu J., Sun X., Gao J., Liu W., Liu J., Chen X., Li J., Wang X. (2025). Effect of Carbon on the Formation of Cobalt Silicide and Thermal Stability for DRAM Application: A Comparative Study on PVD and CVD Methods. IEEE Trans. Electron Devices.

[14] Hamilton J.A., Pugh T., Johnson A.L., Kingsley A.J., Richards S.P. (2016). Cobalt(I) Olefin Complexes: Precursors for Metal–Organic Chemical Vapor Deposition of High Purity Cobalt Metal Thin Films. Inorg. Chem..

[15] Zhang L., Wang T., Wang S., Lu X. (2023). The Role of Dipotassium Ethylenediaminetetraacetic Acid and Potassium Oleate on Chemical Mechanical Planarization Relevant to Heterogeneous Materials of Cobalt Interconnects. Mater. Sci. Semicond. Process..

[16] Franz M., Jäckel L., Hu X., Kaßner L., Thurm C., Rittrich D., Helke C., Schuster J., Daniel M., Stahr F. (2025). Low-Temperature ALD of Metallic Cobalt Using the CoCOhept Precursor: Simulation-Assisted Process Development for Deposition on Temperature Sensitive 3D-Structures. JVST A.

[17] Donnecke S., Paul M., Williams P.J.H., Chan S., Tse V., Sachdeva J., Oliver A.G., McIndoe J.S., Paci I. (2024). Mechanistic Study of the Atomic Layer Deposition of Cobalt: A Combined Mass Spectrometric and Computational Approach. Phys. Chem. Chem. Phys..

[18] Breeden M., Wang V., Spiegelman J., Anurag A., Wolf S.F., Moser D., Kanjolia R.K., Moinpour M., Woodruff J., Nemani S. (2021). Proximity Effects of the Selective Atomic Layer Deposition of Cobalt on the Nanoscale: Implications for Interconnects. ACS Appl. Nano Mater..

[19] Zhang Y., Yuan B., Li L., Wang C. (2020). Edge Electrodeposition Effect of Cobalt Under an External Magnetic Field. J. Electroanal. Chem..

[20] Pan B., Yao Y., Peng L., Zhang Q., Yang Y. (2020). Ultrasound-Assisted Pulse Electrodeposition of Cobalt Films. Mater. Chem. Phys..

[21] Jin L., Lv Y.-J., Ran T.-T., He D., Yang F.-Z. (2025). Molecular Structure with Adsorption Behavior of Butynediol and Its Effects on Cobalt Electrodeposition. Mater. Today Commun..

[22] Brogan L.J., Liu Y., Huie M.M., Reid J.D., Kelly J., Shobha H.k., Huang H., Motoyama K., Hu C. (2019). Improved Copper Damascene Wires Using Direct Plate on Cobalt Process. ECS Meet. Abstr..

[23] Proust M., Judong F., Gilet J.M., Liauzu L., Madar R. (2001). CVD and PVD Copper Integration for Dual Damascene Metallization in a 0.18 Μm Process. Microelectron. Eng..

[24] Vorobyova M., Biffoli F., Giurlani W., Martinuzzi S.M., Linser M., Caneschi A., Innocenti M. (2023). PVD for Decorative Applications: A Review. Materials.

[25] Wang P., Shao Y.-H., Ni Z.-H., Hu C.-F., Qu X.-P. (2022). Low-Temperature Copper–Copper Quasi-Direct Bonding with Cobalt Passivation Layer. AIP Adv..

[26] Jeong B.H., Kim D.W., Lee S.Y., Kim D.S., Lee S.H., Lee S.H., Uematsu M., Kokaze Y., Taura Y., Harada M. (2025). Enhancing Cu Interconnect Reflow in Back-End-of-Line Metal Wiring with Ultrathin Co Liners. Jpn. J. Appl. Phys..

[27] Zanders D., Liu J., Obenlüneschloß J., Bock C., Rogalla D., Mai L., Nolan M., Barry S.T., Devi A. (2021). Cobalt Metal ALD: Understanding the Mechanism and Role of Zinc Alkyl Precursors as Reductants for Low-Resistivity Co Thin Films. Chem. Mater..

[28] Wei C.-C., Chou E., Shih S., Lin S.-M. (2015). Bottom-up Filling of Damascene Trenches with Cobalt By Electroplating Process. Meet. Abstr..

[29] Shen Y., Guo J., Wang L., Han H., Ma Y., Xin B., Wang Z. (2023). Acceleration Mechanism of Triethanolamine in Electroless Bath for Pure Cobalt Deposition. J. Electrochem. Soc..

[30] Guo L., Li S., He Z., Fu Y., Qiu F., Liu R., Yang G. (2024). Electroplated Copper Additives for Advanced Packaging: A Review. ACS Omega.

[31] Zhang M., Chang P., Chen P., Hang T., Li M., Wu Y. (2024). Super-Flat and Uni-Oriented Cobalt Film Electrodeposited by Modulating the Crystal Nucleation and Growth Behavior. Appl. Surf. Sci..

[32] Rigsby M.A., Spurlin T.A., Reid J.D. (2021). Alternative Metals for Advanced Interconnects: Cobalt and Beyond. Meet. Abstr..

[33] Josell D., Wheeler D., Moffat T.P. (2012). Modeling Extreme Bottom-Up Filling of Through Silicon Vias. J. Electrochem. Soc..

[34] Yang L., Radisic A., Deconinck J., Vereecken P.M. (2013). Modeling the Bottom-Up Filling of Through-Silicon Vias through Suppressor Adsorption/Desorption Mechanism. J. Electrochem. Soc..

[35] Josell D., Moffat T.P. (2018). Superconformal Copper Deposition in Through Silicon Vias by Suppression-Breakdown. J. Electrochem. Soc..

[36] Josell D., Moffat T.P. (2013). Extreme Bottom-Up Filling of through Silicon Vias and Damascene Trenches with Gold in a Sulfite Electrolyte. J. Electrochem. Soc..

[37] Josell D., Moffat T.P. (2016). Superconformal Bottom-Up Nickel Deposition in High Aspect Ratio through Silicon Vias. J. Electrochem. Soc..

[38] Josell D., Silva M., Moffat T.P. (2016). Superconformal Bottom-Up Cobalt Deposition in High Aspect Ratio Through Silicon Vias. ECS Trans..

[39] Josell D., Moffat T.P. (2015). Bottom-Up Electrodeposition of Zinc in through Silicon Vias. J. Electrochem. Soc..

[40] Rigsby M., Brogan L., Doubina N., Spurlin T., Zhou J., Reid J. (2017). Superconformal Cobalt Fill Through the Use of Sacrificial Oxidants. Meet. Abstr..

[41] Rigsby M.A., Brogan L.J., Doubina N.V., Liu Y., Opocensky E.C., Spurlin T.A., Zhou J., Reid J.D. (2019). The Critical Role of pH Gradient Formation in Driving Superconformal Cobalt Deposition. J. Electrochem. Soc..

[42] Wu J., Wafula F., Branagan S., Suzuki H., Van Eisden J. (2019). Mechanism of Cobalt Bottom-Up Filling for Advanced Node Interconnect Metallization. J. Electrochem. Soc..

[43] Kiruba M. (2022). Butynediol’s Role Beyond Brightening Additive During Electrodeposition of Cobalt. J. Electrochem. Soc..

[44] Kiruba M., Jeyabharathi C. (2020). Discerning the Oscillatory Electrochemical Response During Electrodeposition of Cobalt in the Presence of But-2-Yne-1,4-Diol. J. Solid State Electrochem..

[45] Wu Y., Ju S., Li F., Zhang M., Ren X., Li M. (2024). Tailoring Crystalline Orientation of Electrodeposited Cobalt by Alkynol Additives. Electrochim. Acta.

[46] Pan B., Zhang Q., Liu Z., Yang Y. (2019). Influence of Butynediol and Tetrabutylammonium Bromide on the Morphology and Structure of Electrodeposited Cobalt in the Presence of Saccharin. Mater. Chem. Phys..

[47] Lu T. (2024). A Comprehensive Electron Wavefunction Analysis Toolbox for Chemists, Multiwfn. J. Chem. Phys..

[48] Lu T., Chen F. (2012). Multiwfn: A Multifunctional Wavefunction Analyzer. J. Comput. Chem..

[49] Borji F., Pour A.N., Karimi J., Izadyar M., Keyvanloo Z., Hashemian M. (2017). The Molecular Adsorption of Carbon Monoxide on Cobalt Surfaces: A Dft Study. Prog. React. Kinet. Mech..

[50] Bai X., Duan Z., Nan B., Wang L., Tang T., Guan J. (2022). Unveiling the Active Sites of Ultrathin Co-Fe Layered Double Hydroxides for the Oxygen Evolution Reaction. Chin. J. Catal..

[51] Yang X., Zhou Q., Wei S., Guo X., Chimtali P.J., Xu W., Chen S., Cao Y., Zhang P., Zhu K. (2024). Anion Additive Integrated Electric Double Layer and Solvation Shell for Aqueous Zinc Ion Battery. Small Methods.

[52] Freitas M.B.J.G., Garcia E.M. (2007). Electrochemical Recycling of Cobalt from Cathodes of Spent Lithium-Ion Batteries. J. Power Sources.

[53] Hamulić D., Milošev I., Lützenkirchen-Hecht D. (2018). The Effect of the Deposition Conditions on the Structure, Composition and Morphology of Electrodeposited Cobalt Materials. Thin Solid Films.

[54] Kang J., Sung M., Byun J., Kwon O.J., Kim J.J. (2020). Proton Sensitive Additive for Cobalt Electrodeposition. J. Electrochem. Soc..

[55] Wang X., Shi G., Shi F.-N., Xu G., Qi Y., Li D., Zhang Z., Zhang Y., You H. (2016). Synthesis of Hierarchical Cobalt Dendrites Based on Nanoflake Self-Assembly and Their Microwave Absorption Properties. RSC Adv..

[56] Doubina N.V., Spurlin T.A., Opocensky E.C., Reid J.D. (2020). The Effect of Thermal Annealing on Cobalt Film Properties and Grain Structure. MRS Adv..

[57] Bron M., Holze R. (2000). Spectroelectrochemical Investigation of the Adsorption and Oxidation of Unsaturated C4-Alcohols. Surf. Sci..

