# Supplementary material for: Superconformal Electrodeposition of Cobalt into Micron-Scale Trench with Alkynol Derivatives
Source: Materials (Basel). 2025 Apr 10;18(8):1747. doi: 10.3390/ma18081747 (PMC12029023; doi:10.3390/ma18081747)
Supplement: Supplementary file 1 [file materials-18-01747-s001.zip › materials-3523365-supplementary.pdf]

Supplementary materials

Superconformal electrodeposition of cobalt into micron-scale trench with alkynol derivatives

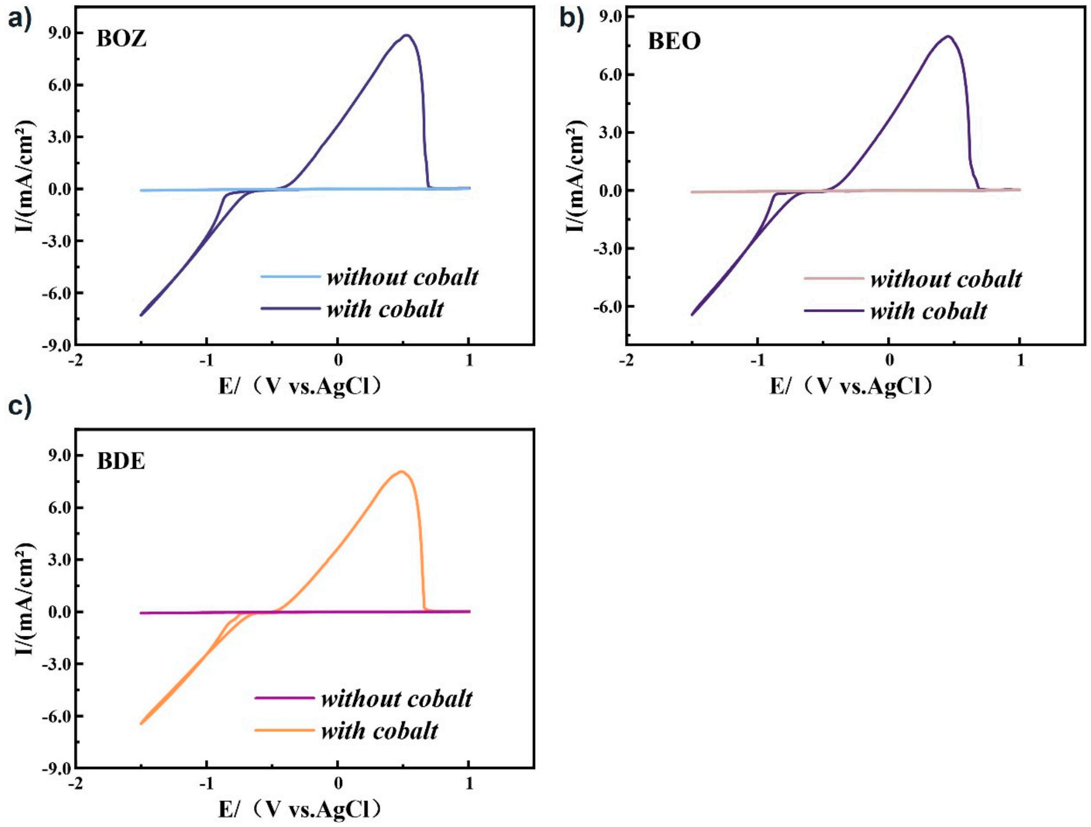

Figure S1. CVs of the additive in different solutions (without cobalt: 30 g/L H_3BO_3 and a pH of 4.01; with cobalt: 0.05 mol/L Co_2SO_4, 30 g/L H_3BO_3 and a pH of 4.00).
